# Mathematical analysis of a within-host model of SARS-CoV-2

**DOI:** 10.1186/s13662-021-03276-1

**Published:** 2021-02-17

**Authors:** Bhagya Jyoti Nath, Kaushik Dehingia, Vishnu Narayan Mishra, Yu-Ming Chu, Hemanta Kumar Sarmah

**Affiliations:** 1 Department of Mathematics, Barnagar College, Sorbhog, 781317 Barpeta, Assam India; 2 Department of Mathematics, Gauhati University, Guwahati, 781014 Assam India; 3 Department of Mathematics, Indira Gandhi National Tribal University, Amarkantak, 484 887 Madhya Pradesh India; 4 Department of Mathematics, Huzhou University, Huzhou, 313000 P.R. China; 5 Hunan Provincial Key Labortory of Mathematical Modeling and Analysis in Engineering, Changsha University of Science & Technology, Changsha, 410114 P.R. China

**Keywords:** 37N25, 34A34, 92B05, SARS-CoV-2, Epithelial cells, Global stability, Basic reproduction number

## Abstract

In this paper, we have mathematically analyzed a within-host model of SARS-CoV-2 which is used by Li et al. in the paper *“The within-host viral kinetics of SARS-CoV-2”* published in (Math. Biosci. Eng. 17(4):2853–2861, 2020). Important properties of the model, like nonnegativity of solutions and their boundedness, are established. Also, we have calculated the basic reproduction number which is an important parameter in the infection models. From stability analysis of the model, it is found that stability of the biologically feasible steady states are determined by the basic reproduction number $(\chi _{0})$. Numerical simulations are done in order to substantiate analytical results. A biological implication from this study is that a COVID-19 patient with less than one basic reproduction ratio can automatically recover from the infection.

## Introduction

A deadly virus, called SARS-CoV-2 and mostly known as coronavirus, has affected most of the countries from November 2019. The World Health Organization declared it as a pandemic, as the virus affected more than a hundred countries and killed lots of people worldwide, especially in Europe and the USA in a short period [1]. During this rampant situation, to stop the virus propagation, almost all the regions of the world have been locked-down by applying strict social distancing measures and banned social gathering in all circumstances. Research is going on all over the world to control this epidemic from different points of view, such as pathology [2, 3], microbiology [4, 5], mathematics [6, 7], and so on [8–10]. Until now, several mathematical models have been suggested to study the population dynamics of coronavirus [8], but few of them could predict the outcomes accurately.

As we know, mathematical studies demonstrate the best strategies for controlling any type of disease or virus infection in humans [11, 12]. The modeling of any disease helps to decide on public health and introduces the disease treatment. When the primary studies of any deadly disease cannot give us any concluding remarks to take significant decisions to control the pandemic situation, the key facts of mathematical modeling always help the human civilization. Mathematical models of viral dynamics with viral and antiviral drug effects are examined and assisted by their transparent structures and biologically feasible parameters. There is much work to be done now and in the future to assess the figure of confirmed cases of COVID-19. Also, a few studies focused on confirmed cases, risk history, and disease schedule characteristics [8, 12, 13]. Mathematical models of epidemiology have been developed to help policymakers in making the right decisions at the right time. These models highlight that isolation plays an important role in controlling the spread of the virus [6, 13]. But this is not a solution to the long-term vision as a country’s economic growth globally is declining due to the locked-downs to maintain social distancing [7]. Therefore, researchers and scientists are now developing dynamic viral within-host models to understand the mechanisms of SARS-CoV-2 in the human body. To date, only a few host-level models [4, 5] have been developed to understand the SARS-CoV-2 replication cycle and its interactions with the immune system. Most of them are linked to HIV [14], hepatitis virus [15], Ebola [16], influenza [9, 17], and other models.

At present, clinically there is no effective treatment developed to remove the virus from the human body. However, the research is going on. Researchers provided many treatments (like plasma therapy, monoclonal antibody therapy, etc.) which are effective for early diseases like SARS-CoV, MERS-CoV, Ebola, Influenza, HIV-like virus disease. Also we all know that our body immune system gives a good response to fight any viruses or diseases [2].

At the time of SARS-CoV-2 infection, macrophages are first targeted by SARS-CoV-2 and after that these SARS-CoV-2 propagate to T cells. At this stage of the virus route, the T cells activate and further they involve differentiation. Besides, the T cells produce cytokines (INF-*α*, IL-6, and IL-10) associated with the different types of a T cell. A large amount of cytokines provides a greater activation of the immune response to fight the virus. Particularly T cells, CD4^+^ T cells, and CD8^+^ T cells have played a significant antiviral role in a fight against pathogens. There is also a risk of mounting autoimmunity or devastating inflammation. CD4^+^ T cells help the immune system of the body by generating virus-specific antibodies with the activation of T-dependent B cells. However, CD8^+^ T cells can kill virally infected cells, as they are cytotoxic. In general, a large number of CD8^+^ T cells in the infected SARS-CoV-2 body are found in nearly 80% of the total infiltrative inflammatory cells in the interstitial pulmonary tract, which play a significant role in clearing CoVs. The loss of CD4^+^ T cells is correlated with reduced conscription of lymphocytes and neutralizing the production of antibodies and cytokines, resulting in severe immune-mediated interstitial pneumonitis and delayed SARS-CoV lung clearance [2, 18].

Researchers have shown that there is a long-lasting and persistent response of T cells to the S and other structural proteins (including the proteins M and N), which provides sufficient knowledge to draft SARS vaccine by combining viral structural proteins. These types of vaccine may provide a strong, efficient, and long-term response to the virus by memory cells [3]. Also, clinical trials show that a monoclonal antibody therapy is an effective treatment tool which responds better to SARS-CoV-2 [10]. In our paper, we studied a model of in-host viral kinetics, that describes the response of SARS-CoV-2 to epithelial cells. In Sect. 2, we describe a previously proposed model by Li et al. [5]. We have shown in Sect. 3 that the solutions of the model are biologically feasible. The basic reproduction number is computed and steady state analysis is done in Sect. 4. Sections 5 and 6 deal with the local and global stability of the model, respectively. In Sect. 7, we fitted our theoretical results with MATLAB. A concluding discussion is presented in Sect. 8.

## The SARS-CoV-2 infection model

To study the within-host dynamics of SARS-CoV-2 infection, we consider the mathematical model used by Li et al. [5], which is represented by the following ordinary differential equations system:

2.1 $$ \begin{aligned} &\frac{dE_{p}(t)}{dt}=d_{E} \bigl(E_{p}(0)-E_{p}(t)\bigr)-\beta E_{p}(t)v(t), \\ &\frac{dE^{*}_{p}(t)}{dt}=\beta E_{p}(t)v(t)-d_{E^{*}}E_{p}^{*}(t), \\ &\frac{dv(t)}{dt}=\pi _{v}E_{p}^{*}(t)-d_{v}v(t). \end{aligned} $$

with the initial conditions as below:

2.2 $$ E_{p}(0)>0,\qquad E_{p}^{*}(0)\geq 0\quad \text{and}\quad v(0)\geq 0. $$

Here, the model consists of three population compartments: virus-free pulmonary epithelial cells denoted by $E_{p}(t)$, virus infected pulmonary epithelial cells denoted by $E^{*}_{p}(t)$, and the free virus denoted by $v(t)$. Also, $d_{E}, d_{E^{*}}$, and $d_{v}$ represent the death rates of virus-free epithelial cells, virus infected epithelial cells, and the free virus, respectively. Also *β* denotes the rate at which the virus-free epithelial cells are infected by free virus, $\pi _{v}$ is the production rate of free virus. In this model, the term $d_{E}E_{p}(0)$ describes the constant regeneration rate of the virus-free pulmonary epithelial cells.

## Basic properties of model (2.1)

### Lemma 1

*All analytic solutions of model* (2.1) *with initial conditions* (2.2) *are nonnegative for all* $t>0$.

### Proof

We can write the first equation of model (2.1) as

3.1 $$ \frac{d}{dt} \biggl[E_{p}(t)\operatorname{exp} \biggl\lbrace \int _{0}^{t} \bigl(\beta v(\mu )+d_{E} \bigr)\,d\mu \biggr\rbrace \biggr]=d_{E}E_{p}(0) \operatorname{exp} \biggl\lbrace \int _{0}^{t} \bigl(\beta v(\mu )+d_{E} \bigr)\,d\mu \biggr\rbrace .$$

Therefore,

$$ E_{p}(t)\operatorname{exp} \biggl\lbrace \int _{0}^{t}\beta v(\mu )\,d\mu +d_{E}t \biggr\rbrace -E_{p}(0)= \int _{0}^{t} \biggl[d_{E}E_{p}(0) \operatorname{exp} \biggl\lbrace \int _{0}^{u}\beta v(\mu )\,d\mu +d_{E}u \biggr\rbrace \biggr]\,du, $$

which implies

3.2 $$\begin{aligned} E_{p}(t)={}&\operatorname{exp} \biggl\lbrace - \int _{0}^{t}\beta v(\mu )\,d\mu -d_{E}t \biggr\rbrace \\ &{}\times \biggl[E_{p}(0)+ \int _{0}^{t} \biggl[d_{E}E_{p}(0) \operatorname{exp} \biggl\lbrace \int _{0}^{u}\beta v(\mu )\,d\mu +d_{E}u \biggr\rbrace \biggr]\,du \biggr]. \end{aligned}$$

From the above equation, it is clear that $E_{p}(t)>0$ for $t>0$. It can be shown in the same way that $E_{p}^{*}(t)\geq 0, v(t)\geq 0$ for $t>0$ [19]. □

### Lemma 2

*The solutions* $E_{p}(t), E_{p}^{*}(t),v(t)$ *of the model* (2.1) *are bounded for all* $t>0$.

### Proof

Adding the first two equations of the model (2.1), we get

$$ \frac{d}{dt} \bigl\lbrace E_{p}(t)+E_{p}^{*}(t) \bigr\rbrace =d_{E}\bigl(E_{p}(0)-E_{p}(t) \bigr)-d_{E^{*}}E_{p}^{*}(t) \leq d_{E}E_{p}(0)- \delta \bigl\lbrace E_{p}(t)+E_{p}^{*}(t)\bigr\rbrace , $$

where *δ*=min$\lbrace d_{E},d_{E^{*}}\rbrace $. Therefore, the numbers of both the virus-free and infected pulmonary epithelial cells are always bounded. From the third equation of the model, we can easily show that the population of the free virus *v* is also bounded above. We consider $K \geq 0$ as the maximum of the above bounds. Therefore, we get the bounded set

$$ \Omega =\biggl\lbrace E_{p}(t),E_{p}^{*}(t),v(t) \in \mathbb{R}^{3}:0\leq E_{p}(t)+E_{p}^{*}(t) \leq \frac{d_{E}E_{p}(0)}{\delta }, v\leq K\biggr\rbrace , $$

where $K\geq 0$. It is clear that this set is convex. Also using the nonnegativity criteria of solutions of the model (2.1) from Lemma 1 and the boundedness conditions from Lemma 2, it is clear that with the initial conditions $(E_{p}(0), E_{p}^{*}(0), v(0) )\in \Omega $, we have solutions of the model (2.1) again in Ω, i.e., $(E_{p}(t), E_{p}^{*}(t), v(t) )\in \Omega $ for all $t\geq 0$. Hence, the set Ω is positively invariant w.r.t. the model (2.1). □

## Basic reproduction number and steady states

It is straightforward to show that the system has a noninfected steady state $S_{1}(E_{p}(0),0,0)$. Now, the basic reproduction ratio of the model (2.1) will be calculated with the help of the next generation matrix method. Considering $Y=(E_{p}^{*}(t),v(t),E_{p}(t))$, we can write the system (2.1) as

4.1 $$ \frac{dY}{dt}=A(Y)-T(Y). $$

Here $A(Y)$ stands for the rate of new infections, $T(Y)$ stands for the transfer rate of individuals; they are given by

$$ A(Y)= \begin{pmatrix} \beta E_{p}(t)v(t) \\ 0 \\ 0 \end{pmatrix} \quad\text{and}\quad T(Y)= \begin{pmatrix} d_{E^{*}}E_{p}^{*}(t) \\ d_{v}v(t)-\pi _{v}E_{p}^{*}(t) \\ \beta E_{p}(t)v(t)-d_{E}(E_{p}(0)-E_{p}(t)) \end{pmatrix} . $$

The jacobian matrices $DA(E_{p}(0))$ and $DT(E_{p}(0))$ at the noninfected steady state are

$$ DA\bigl(E_{p}(0)\bigr)= \begin{pmatrix} a_{2 \times 2} & 0_{2\times 1} \\ 0_{1\times 2} & 0 \end{pmatrix} ,\qquad DT\bigl(E_{p}(0)\bigr)= \begin{pmatrix} t_{2 \times 2}& & 0_{2\times 1} \\ 0 & \beta E_{p}(0) & d_{E} \end{pmatrix}, $$

where

$$ a_{2\times 2}= \begin{pmatrix} 0 & \beta E_{p}(0) \\ 0 & 0 \end{pmatrix} ,\qquad t_{2 \times 2}= \begin{pmatrix} d_{E^{*}} & 0 \\ -\pi _{v} & d_{v} \end{pmatrix} . $$

Now,

$$ at^{-1}=\frac{1}{d_{E^{*}}d_{v}} \begin{pmatrix} \pi _{v}\beta E_{p}(0) & d_{E^{*}}\beta E_{p}(0) \\ 0 & 0 \end{pmatrix} . $$

Therefore, the basic reproduction number $(\chi _{0})$ of the system (2.1) is given by the spectral radius of the matrix $at^{-1}$ and hence $\chi _{0}=\frac{\pi _{v}\beta E_{p}(0)}{d_{E^{*}}d_{v}}$.

Also, from a simple calculation it is clear that the model (2.1) has a unique infected steady state

$$ S_{2}=\bigl(\bar{E_{p}},\bar{E_{p}^{*}}, \bar{v}\bigr) \quad\text{whenever } \chi _{0}>1, $$

where

$$\begin{aligned} &\bar{E_{p}}=\frac{d_{E^{*}}d_{v}}{\beta \pi _{v}}=E_{p}(0) \frac{1}{\chi _{0}}, \\ &\bar{E_{p}^{*}}=\frac{d_{v}\bar{v}}{\pi _{v}}= \frac{d_{v}d_{E}}{\beta \pi _{v}}( \chi _{0}-1), \\ &\bar{v}=\frac{d_{E}}{\beta }(\chi _{0}-1). \end{aligned}$$

## Local stability

In this section, we study the local stability behaviors at the different steady states of the model.

### Theorem 1

*If* $\chi _{0}<1$, *then the noninfected steady state* $S_{1}$ *is locally asymptotically stable in* Ω *while for* $\chi _{0}>1$ *it becomes unstable*.

### Proof

For the noninfected steady state $S_{1}$, the Jacobian matrix $J(S_{1})$ is given by

5.1 $$ J(S_{1})= \begin{pmatrix} -d_{E} & 0 & -\beta E_{p}(0) \\ 0 & -d_{E^{*}} & \beta E_{p}(0) \\ 0 & \pi _{v} & -d_{v} \end{pmatrix} .$$

The characteristic equation of the matrix $J(S_{1})$ is

5.2 $$ \lambda ^{3}+a_{1}\lambda ^{2}+a_{2} \lambda +a_{3}=0, $$

where

$$\begin{aligned} &a_{1}= d_{E}+d_{E^{*}}+d_{v}, \\ &a_{2}= d_{E}(D_{E^{*}}+d_{v})+d_{E^{*}}d_{v}(1- \chi _{0}), \\ &a_{3}= d_{E}d_{E^{*}}d_{v}(1-\chi _{0}), \\ &a_{1}a_{2}-a_{3}=d_{E}^{2}(d_{E^{*}}+d_{v})+d_{E}(d_{E^{*}}+d_{v})^{2}+d_{E^{*}}d_{v}(d_{E^{*}}+d_{v}) (1- \chi _{0}). \end{aligned}$$

Therefore, if $\chi _{0}<1$, then all the conditions of the Routh–Hurwitz criterion are satisfied. Hence, the noninfected steady state $S_{1}$ becomes locally asymptotically stable if $\chi _{0}<1$ and unstable if $\chi _{0}>1$. □

### Theorem 2

*The virus*-*infected steady state* $S_{2}$ *is locally asymptotically stable whenever* $\chi _{0}>1$.

### Proof

For the infected steady state $S_{2}$, the Jacobian matrix $J(S_{2})$ is given by

5.3 $$ J(S_{2})= \begin{pmatrix} -d_{E}-\beta \bar{v} & 0 & -\beta E_{p} \\ \beta \bar{v} & -d_{E^{*}} & \beta E_{p} \\ 0 & \pi _{v} & -d_{v} \end{pmatrix} .$$

The characteristic equation of the above matrix becomes

5.4 $$ \lambda ^{3}+b_{1}\lambda ^{2}+b_{2} \lambda +b_{3}=0, $$

where

$$\begin{aligned} &b_{1}=d_{E}+\beta \bar{v}+d_{E^{*}}+d_{v}, \\ &b_{2}=(d_{E}+\beta \bar{v}) (d_{E^{*}}+d_{v}), \\ &b_{3}=\beta \bar{v}d_{E^{*}}d_{v}, \\ &b_{1}b_{2}-b_{3}=(d_{E}+\beta \bar{v}+d_{E^{*}}) (d_{E}+\beta \bar{v}) (d_{E^{*}}+d_{v})+d_{v}d_{E}(d_{E^{*}}+d_{v})+d_{v}^{2} \beta \bar{v}. \end{aligned}$$

Thus, $b_{1}>0, b_{2}>0, b_{3}>0$, and $b_{1}b_{2}-b_{3}>0$ whenever $\chi _{0}>1$. This implies that all conditions of the Routh–Hurwitz criterion are satisfied. Hence, the infected steady state $S_{2}$ is locally asymptotically stable whenever it exists. □

## Global stability

From local stability analysis, the behaviors of the system (2.1) near the steady states are obtained. For the behavior of the system (2.1) far away from the steady states, we have carried out a global stability analysis in this section.

### Theorem 3

*If* $\chi _{0}\leq 1$, *then the noninfected steady state* $S_{1}$ *approaches a globally asymptotically stable state in* Ω *and it becomes unstable if* $\chi _{0}>1$.

### Proof

Define Lyapunov functional of the model (2.1) as

6.1 $$ L=\frac{\pi _{v}}{d_{E^{*}}}E_{p}^{*}+v. $$

Now, we differentiate w.r.t. time to obtain

6.2 $$ \frac{dL}{dt}=d_{v}v \biggl( \frac{\pi _{v}\beta E_{p}}{d_{E^{*}}d_{v}}-1 \biggr)\leq d_{v}v (\chi _{0}-1 ). $$

It is clear from (6.2) that when $\chi _{0}\leq 1$, $\frac{dL}{dt}\leq 0$. Define *S* to represent the set of solutions of model (2.1) where $\frac{dL}{dt}=0$. We have $\frac{dL}{dt}=0$ when $v=0$ or $\chi _{0}=1$ and $E_{p}\leq E_{p}(0)$. Using Lyapunov–Lasalle theorem [20], we have all curves in Ω approach the set *S* which is also positively invariant. Again, on the boundary of Ω where $v=0$, we have $E_{p}^{*}=0$ and $\frac{dE_{p}(t)}{dt}=d_{E}(E_{p}(0)-E_{p}(t))$. Hence, $E_{p}\rightarrow E_{p}(0)$ as $t\rightarrow \infty $. Therefore, when $\chi _{0}\leq 1$, all solution curves in the domain Ω go to the virus-free steady state $S_{1}$ and hence $S_{1}$ is globally asymptotically stable.

From $J(S_{1})$, it can be easily seen that for $\chi _{0}>1$, one root of the characteristic equation will be positive. Hence, the noninfected steady state $S_{1}$ is unstable when $\chi _{0}>1$. □

Now, the global behavior of the virus-infected steady state $S_{2}$ whenever it exists will be studied by applying Li and Muldowney criterion [21]. It is easy to visualize the simple connectedness of the interior of set Ω. Also, for $\chi _{0}>1$, the system (2.1) has the unique steady state $S_{2}$ in $\mathrm{int}(\Omega )$. Theorem 3 verifies that $H\subset \Omega $ exist where *H* is an absorbing compact set for model (2.1). Hence, all the assumptions for Li and Muldowney global stability criterion [21] are satisfied, and we have the following theorem.

### Theorem 4

*The infected steady state* $S_{2}$ *is globally asymptotically stable whenever* $R_{0}>1$.

### Proof

The Jacobian matrix of the model (2.1) is

6.3 $$ J= \begin{pmatrix} -d_{E}-\beta v & 0 & -\beta E_{p} \\ \beta v & -d_{E^{*}} & \beta E_{p} \\ 0 & \pi _{v} & -d_{v} \end{pmatrix} .$$

The associated second compound matrix $J^{[2]}$ [21, 22] is given by

6.4 $$ J^{[2]}= \begin{pmatrix} -d_{E}-d_{E^{*}}-\beta v & \beta E_{p} & \beta E_{p} \\ \pi _{v} & -d_{E}-d_{v}-\beta v & 0 \\ 0 & \beta v & -d_{E^{*}}-d_{v} \end{pmatrix} .$$

Defining

$$ W= \begin{pmatrix} 1 & 0 & 0 \\ 0 & \frac{E_{p}^{*}}{v} & 0 \\ 0 & 0 & \frac{E_{p}^{*}}{v} \end{pmatrix} , $$

to compute the matrix $W_{f}$, each entry $w_{ij}$ of matrix *W* has to be replaced by its derivative in the direction of *f*. Then,

$$ W_{f}W^{-1}= \begin{pmatrix} 0 & 0 & 0 \\ 0 & \frac{{E_{p}^{*}}'}{E_{p}^{*}}-\frac{v{'}}{v} & 0 \\ 0 & 0 & \frac{{E_{p}^{*}}'}{E_{p}^{*}}-\frac{v{'}}{v} \end{pmatrix} $$

and

$$\begin{aligned} B&=W_{f}W^{-1}+WJ^{[2]}W^{-1} \\ &= \begin{pmatrix} -d_{E}-d_{E^{*}}-\beta v & \beta E_{p}\frac{v}{E_{p}^{*}} & \beta E_{p} \frac{v}{E_{p}^{*}} \\ \pi _{v}\frac{E_{p}^{*}}{v} & \frac{{E_{p}^{*}}'}{E_{p}^{*}}- \frac{v{'}}{v}-d_{E}-d_{v}-\beta v & 0 \\ 0 & \beta v & \frac{{E_{p}^{*}}'}{E_{p}^{*}}-\frac{v{'}}{v}-d_{E^{*}}-d_{v} \end{pmatrix} \\ &= \begin{pmatrix} B_{11} & B_{12} \\ B_{21} & B_{22} \end{pmatrix}, \end{aligned}$$

where $B_{11}=(-d_{E}-d_{E^{*}}-\beta v),B_{12}=\left(\begin{array}{cc} \beta E_{p}\frac{v}{E_{p}^{*}} & \beta E_{p}\frac{v}{E_{p}^{*}} \end{array}\right),B_{21}=\left(\begin{array}{c} \pi_{v}\frac{E_{p}^{*}}{v}\\ 0 \end{array}\right)$, and

$$ B_{22}= \begin{pmatrix} \frac{{E_{p}^{*}}'}{E_{p}^{*}}-\frac{v{'}}{v}-d_{E}-d_{v}-\beta v & 0 \\ \beta v & \frac{{E_{p}^{*}}'}{E_{p}^{*}}-\frac{v{'}}{v}-d_{E^{*}}-d_{v} \end{pmatrix} . $$

Now, define Lozinskii measure as follows:

6.5 $$ \mu (B)\leq \operatorname{max}\lbrace g_{1}.g_{2}\rbrace, $$

where $g_{1}=\mu (B_{11})+|B_{12}|$ and $g_{2}=|B_{21}|+\mu (B_{22})$. Here, $|B_{12}|$ and $|B_{21}|$ are with respect to vector norm.

Now,

$$\begin{aligned} &\mu (B_{11})=-d_{E}-d_{E^{*}}-\beta v, \\ &\mu (B_{22})=\max \biggl\lbrace \frac{{E_{p}^{*}}'}{E_{p}^{*}}- \frac{v{'}}{v}-d_{E}-d_{v}-\beta v+\beta v, \frac{{E_{p}^{*}}'}{E_{p}^{*}}-\frac{v{'}}{v}-d_{E^{*}}-d_{v} \biggr\rbrace \\ &\phantom{\mu (B_{22})}=\frac{{E_{p}^{*}}'}{E_{p}^{*}}-\frac{v{'}}{v}-\delta -d_{v} \quad\text{where } \delta =\operatorname{min}\lbrace d_{E}, d_{E^{*}}\rbrace, \\ &\vert B_{12} \vert =\beta E_{p}\frac{v}{E_{p}^{*}}, \quad\text{and}\quad \vert B_{21} \vert = \pi _{v} \frac{E_{p}^{*}}{v}. \end{aligned}$$

Using model equations,

$$ \frac{{E_{p}^{*}}'}{E_{p}^{*}}=\frac{\beta E_{p}v}{E_{p}^{*}}-d_{E^{*}} \quad\text{and}\quad \frac{v{'}}{v}=\frac{\pi _{v}E_{p}^{*}}{v}-d_{v}. $$

Hence,

$$\begin{aligned} &g_{1}=\frac{{E_{p}^{*}}'}{E_{p}^{*}}-d_{E}-\beta v, \\ &g_{2}=\frac{{E_{p}^{*}}'}{E_{p}^{*}}-\delta. \end{aligned}$$

Therefore, we get

$$ \mu (B)\leq \frac{{E_{p}^{*}}'}{E_{p}^{*}}-\delta $$

for large *t*. Let $(E_{p}(t), E_{p}^{*}(t), v(t))$ be an arbitrary solution such that $(E_{p}(0), E_{p}^{*}(0), v(0))\in H$ and consider any solution $(E_{p}(t), E_{p}^{*}(t), v(t))\in H$ for all $t\geq \bar{t}$ where *t̄* is a sufficiently large time. Then, for $t > \bar{t}$,

6.6 $$ \frac{1}{t} \int _{0}^{t}\mu (B)\,ds \leq \frac{1}{t} \int _{0}^{ \bar{t}}\mu (B)\,ds+\frac{1}{t}\ln \frac{E_{p}^{*}(t)}{E_{p}^{*}(\bar{t})}- \biggl(\frac{t-\bar{t}}{t} \biggr)\delta. $$

Consequently,

$$ \bar{q_{2}}:=\lim_{t\rightarrow \infty }\sup \sup _{x_{0}\in H} \frac{1}{t} \int _{0}^{t}\mu \bigl(B\bigl(x(s,x_{0}) \bigr)\bigr)\,ds< 0. $$

 □

## Numerical simulation

This section is devoted to numerical simulations in order to substantiate the analytic results. Using Matlab software, analytic results are fitted with the parameters from biologically feasible range for SARS-CoV-2.

**Figure 1:**
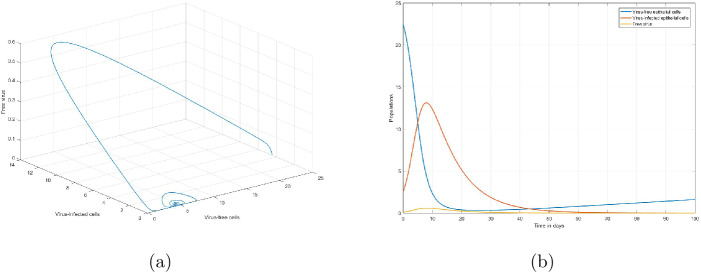
Dynamics of the numbers of virus-free epithelial cells, virus-infected epithelial cells, and SARS-CoV-2 virus when $\chi _{0}>1$

Based on chest radiograph score data, Li et al. [5] have estimated the parameters of the model (2.1) using the Monte Carlo Markov Chain (MCMC) method. Using that parameter set, more specifically, $E_{p}(0)=22.41, E_{p^{*}}(0)=2.59, v(0)=0.061, \pi _{v}=0.24, \beta =0.55, d_{E}=10^{-3}, d_{E^{*}}=0.11$, and $d_{v}=5.36$, we have $\chi _{0}=5.01716>1$. Theorem 4 implies that the infected equilibrium point $S_{2}(4.467,0.163, 0.007)$ is globally asymptotically stable. Figure 1(a) verifies this analytic result. Also, Fig. 1(b) describes the dynamics the numbers of virus-free epithelial cells, virus infected epithelial cells, and free virus.

**Figure 2:**
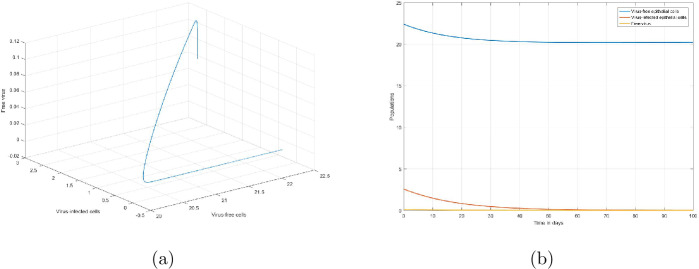
Dynamics of the numbers of virus-free epithelial cells, virus-infected epithelial cells, and SARS-CoV-2 virus when $\chi _{0}<1$

In order to verify the analytic results of the noninfected steady point $S_{1}$, we consider lowering the infection rate from *β* to 0.1*β* by implementing a drug [5]. Hence with this assumption, considering the other parameters the same as in the above simulation, we get $\chi _{0}=0.501716<1$. As stated by Theorem 3, the noninfected steady state $S_{1}(22.41, 0, 0)$ is globally asymptotically stable. This analytic result can be verified by Fig. 2(a). Also, the dynamics of the numbers of virus-free epithelial cells, virus-infected epithelial cells, and virus in this scenario are depicted by Fig. 2(b).

In the above simulations, we have considered that infection rate *β* can be lowered by implementing a drug, which leads to the removal of the virus from the human body. But with greater infection rate and other parameters in such a way that $\chi >1$, the viral load cannot be controlled and infection persists in the human body. This situation can be handled with a proper treatment strategy, otherwise the number of the virus-free epithelial cells will decrease and those of the virus-infected epithelial cells and the free virus will increase in a patient’s body with time. As a result, the patient’s condition will deteriorate with time, which will lead to extreme conditions like death of the patient.

## Discussion

In this paper, we have considered a basic model for within-host dynamics of SARS-CoV-2 used by Li et al. [5] and mathematically analyzed it. First of all, we have proved that all analytic solutions of model (2.1) are nonnegative and uniformly bounded, conditions which are necessary for a biologically feasible model. For the model, we have found two biologically feasible steady states, noninfected and infected steady states. Local stability of both steady states was discussed. Also, we have found the global stability criteria for both steady states. Biologically, it follows that for the basic reproduction number $\chi _{0}<1$, infection will be cleared from a human body without any treatment; otherwise we have to implement some treatment in order to reduce and to remove the infection from the body. It is our future work to apply different treatment policies in this model in order to clear the virus from an infected human body. It is also found that if basic reproduction number is greater than one then infection will persist for any amount of viral load in the host’s body.

## Data Availability

Not applicable.
